# Rho GTPases as Key Molecular Players within Intestinal Mucosa and GI Diseases

**DOI:** 10.3390/cells10010066

**Published:** 2021-01-04

**Authors:** Rashmita Pradhan, Phuong A. Ngo, Luz d. C. Martínez-Sánchez, Markus F. Neurath, Rocío López-Posadas

**Affiliations:** Department of Medicine 1, University of Erlangen-Nuremberg, 91054 Erlangen, Germany; Rashmita.Pradhan@uk-erlangen.de (R.P.); Phuong.NgoAnh@uk-erlangen.de (P.A.N.); LuzdelCarmen.MartinezSanchez@uk-erlangen.de (L.d.C.M.-S.); markus.neurath@uk-erlangen.de (M.F.N.)

**Keywords:** Rho GTPases, intestine, inflammation, cancer, intestinal epithelial cells, T cells

## Abstract

Rho proteins operate as key regulators of the cytoskeleton, cell morphology and trafficking. Acting as molecular switches, the function of Rho GTPases is determined by guanosine triphosphate (GTP)/guanosine diphosphate (GDP) exchange and their lipidation via prenylation, allowing their binding to cellular membranes and the interaction with downstream effector proteins in close proximity to the membrane. A plethora of in vitro studies demonstrate the indispensable function of Rho proteins for cytoskeleton dynamics within different cell types. However, only in the last decades we have got access to genetically modified mouse models to decipher the intricate regulation between members of the Rho family within specific cell types in the complex in vivo situation. Translationally, alterations of the expression and/or function of Rho GTPases have been associated with several pathological conditions, such as inflammation and cancer. In the context of the GI tract, the continuous crosstalk between the host and the intestinal microbiota requires a tight regulation of the complex interaction between cellular components within the intestinal tissue. Recent studies demonstrate that Rho GTPases play important roles for the maintenance of tissue homeostasis in the gut. We will summarize the current knowledge on Rho protein function within individual cell types in the intestinal mucosa in vivo, with special focus on intestinal epithelial cells and T cells.

## 1. Gut

The intestine, or informally referred as “gut”, is the primary digestive organ in the body. In order to fulfil its main function (nutrient/water absorption), an enlarged length and a special circular folding structure of the mucosal layer contribute to the extension of the absorptive surface of the small intestine [1]. Upon nutrient absorption there, the large intestine takes charge of the remaining content from the ileum, and contributes to the absorption of water and ions, but no chemical digestion occurs there. In contrast, the colon has a very developed luminal bacterial flora, which participates in the digestion and synthesis of essential vitamins for the body, like vitamin K. Being the terminal part of the gastrointestinal tract, the colon mucosa layer lacks circular folds and villi, which enables the formation and elimination of feces [2]—the final step in the digestive process.

### 1.1. Cellular Players within the Intestinal Mucosa

In the gut, the human body is separated from the environment (intestinal lumen) by a mucosa layer, which itself consists of three sublayers: the epithelium, lamina propria and lamina muscularis mucosae. The epithelial layer covers the whole mucosa facing directly to the lumen, and protecting against the invasion of harmful agents. The main function of the gut, nutrition and water absorption, is carried out by the epithelial layer. Various immune and stroma cells located in the lamina propria, such as fibroblasts, lymphocytes, innate lymphoid cells, plasma cells, macrophages, eosinophilic leukocytes and mast cells, play key roles to keep an immunological equilibrium at the intestinal mucosa for the maintenance of tissue homeostasis [3]. The muscularis mucosae is a smooth and thin muscle layer, which generates the movement and folding of the mucosa to permit peristalsis. Together, this complex multicellular system along the intestinal tract represents the most extended immune organ of the body [4].

Cellular complexity is even patent within the intestinal epithelium. Located at the crypt bottom, stem cells are responsible for cell proliferation, giving rise to daughter cells, which in turn differentiate into various Intestinal Epithelial Cell (IEC) types. Post-mitotic cells move upwards to the villus tip (migration), where aged cells are extruded in order to balance cell numbers and exclude potentially damaged cells. In the villus, absorptive cells (enterocytes) covering most part of the epithelial layer coexist with Goblet, Enteroendocrine and Tuft cells, while Paneth cells are restricted to the crypt base, intercalated between stem cells. In the small intestine mucosa, enterocytes are responsible for nutrient digestion and absorption. Located between enterocytes, goblet cells secret several mucine molecules that create a gel-like layer, protecting the epithelium from direct contact with bacteria in the lumen [5]. Paneth cells secrete microbicide granules containing α-defensins, C-type lectins and lysozyme into the lumen upon detection of signals from bacteria [6], but also essential growth factors like EGF, Wnt3, TGF-β inducing stem cell proliferation, contributing to the homeostatic epithelial self-renewal [7]. Enteroendocrine cells (EECs) account for less than 1% of the intestinal epithelial population, nonetheless they compose one of the largest endocrine organs in the body [8]. They are specialized in producing hormones and signaling molecules upon sensing of luminal nutrients, in order to regulate gut peristalsis, food intake and digestion. Last but not least, tuft cells are chemosensory epithelial cells of the intestine; they have brush-like microvilli and possess a taste signaling apparatus which enables them to act as sensors triggering biological responses by secreting activated mediators. They are known as the primary source of interleukin-25 in the intestine, which is important to promote type 2 immunity in defense from parasitic infection [9]. Similar to the small intestine, the large intestine mucosa also consists of enterocytes and goblet cells, as well as EEC and Tuft cells, but lack Paneth cells [10]. While the enterocytes absorb water, vitamins, salt and ions, the mucus layer produced by goblet cells not only protects the epithelium but also supports the movement of feces through the colon.

In conclusion, intestinal mucosa is a very complex system, with several layers and a multicellular composition. Alterations of this complex system at the mucosa can lead to pathological conditions, such as chronic intestinal inflammation or cancer.

### 1.2. IBD

Inflammatory Bowel Disease (IBD) is a chronic intestinal disorder group involving ulcerative colitis (UC) and Crohn’s disease (CD) [11]. IBD symptoms vary depending on inflammation severity and location, and include diarrhea, fever, fatigue, abdominal pain, bloating and cramping, blood in stool, reduced appetite and weight loss. IBD is considered a multifactorial disease resulting from an exacerbated immune reaction against components of the intestinal microbiota in a genetically predisposed individual. There are many external factors that increase the risk of developing IBD or aggravate IBD symptoms, such as age, smoking, stress, westernized diet, etc. Even though IBD is a common and lifelong rather than a life-threatening disease, both UC and CD lead to serious complications like colon cancer, blood clots, medication side effects or primary sclerosing cholangitis. Despite the fact that IBD patients can have full life expectancy, patients with an age over 50 still have a high mortality rate due to those colitis-associated complications [12]. Although IBD therapeutics are developing tremendously, still no causative treatment is currently available [13].

Together with uncontrolled activation of immune cells, many studies have proven that development of gut inflammatory disorders such as IBD are associated with marked alterations of IECs, leading to increased tight junction permeability and altered cytoskeletal rearrangement [14]. Thus, protecting epithelial homeostasis reduces inflammation development in IBD [15]. Epithelial integrity is regulated by the turnover process, which in turn highly depends on the actin cytoskeleton. Therefore, epithelial homeostasis is tightly linked to cytoskeletal regulation and cell–cell tight junctions (TJs) proteins [16]. In this context, Rho GTPases, as key regulators of the cytoskeleton, are known to be critically involved in the regulation of intestinal epithelial barrier functions. Dysregulation of Rho protein function could alter actomyosin contractility, and impair barrier function within epithelial cells [14]. Along with Intestinal epithelial cells (IECs) and among other immune cells, T cells also play an essential role in the immune surveillance and maintenance of intestinal homeostasis in the gut. Likewise, Rho GTPases play an important role in multiple T-cell functions, including cell polarization and migration, immune synapse formation, and modulation of T-cell receptor (TCR) signaling [17]. Moreover, communication between T cells and IECs is critical for maintenance of epithelial integrity. Together, we can state that Rho-mediated control of the cytoskeletal function in several cell types within the gut mucosa play key roles for the maintenance of tissue homeostasis and avoid the development of local immune reactions which can lead to chronic intestinal inflammation, such as in IBD.

### 1.3. CRC

Colorectal cancer represents one of the most deadly cancer diseases. Mostly it starts as a polyp in the inner lining of the large intestine (adenomatous polyps), which can further develop into cancer (carcinoma). The most widely accepted hypothesis for the origin of CRC is the occurrence of spontaneous mutations within IECs, mostly on the stem cell compartment [18], which then are promoted by multiple factors such as food intake, smoking, age, inflammation, etc. On the other hand, immune surveillance [19] as well as pro-tumor function of different cell types within the tumor microenvironment (TME) [20], such as the blood vessels, immune cells, fibroblasts and the extracellular matrix (ECM) determine the growth and evolution of tumor cells, and therefore the outcome of the cancer disease.

In 1990, a genetic model was introduced by Eric R. Fearon and Bert Volgelstein to explain colorectal tumorigenesis, in which three steps can be described: initiation, promotion and progression [21]. Initially, colorectal tumor formation requires the activation of oncogenes (*RAS* genes, adenomatous polyposis *APC* gene) together with the inactivation of tumor suppressor genes (*TP53*). Sinergy between K-*RA*S and *APC* mutations allows hyperproliferation of tumor cells [22]. The hyperactive mutated *RAS* promotes cell proliferation through the EGFR–RAS–RAF–ERK–JUN/FOS pathway [23], while *APC* mutations leading to activation of Wnt-β-catenin pathway appear as the earliest detectable and most frequent abnormality in CRC tissue [24,25]. β-CATENIN accumulation in the nucleus has found to increase constantly during CRC progression, while the *TP53* gene is well described as “gatekeeper” for cell growth proliferation in several studies [26]. At early stages, only 7% of adenomas had more than one of the mentioned mutations, and this percentage increased significantly along with their development (49% at late progression). However, it is important to note that all the mutations mentioned above were not adequate to promote progression to malignancy stage, and in most cases, cancer formation (carcinomas) requires at least one more allelic loss [21].

IBD patients are accounted as high-risk group for CRC development; it is believed that sustained inflammation acts as pro-tumorigenic factor in CRC. In fact, colitis-associated colorectal cancer (CAC) is considered one of the most severe complication in IBD patients and responsible for 10–15% of IBD deaths [27]. CAC arises at the site of active inflammation, owning to the mutation accumulation in intestinal epithelial cells [27]. CAC has different clinical and molecular characteristics and occurs in younger patients in comparison with sporadic CRC [28]. This demonstrates the tight link between IBD and CRC, which argue for potential overlapping molecular features in their pathological mechanisms.

The substantial oncogenic function of GTPases was first noticed when activation of the rat sarcoma oncogene homolog (*RAS*) was found to be associated with tumorigenesis almost 40 years ago [29]. In humans, *RAS* mutations were identified in 30% to 50% of colorectal cancers [30,31]. Among *RAS* mutations, *K-RAS* was most frequently found (up to 40% in colon tumors) [30] and has been considered as target drug therapy for colorectal cancer. Besides RAS, the involvement of RHO protein function in cancer has been receiving more and more attention in the last decades. Many studies indicated that the expression of these proteins, including RHOA, RHOC, RAC1, RAC2 and CDC42 are upregulated and/or their activities are dysregulated through GEFs, GAPs or GDIs in tumor tissue [32,33,34]. Despite all these novel studies, it remains unclear whether mutation/inhibition or activation of Rho GTPases contribute to tumorigenesis; still controversial is the fact that some Rho GTPase effectors and pathways are oncogenic while others act as tumor suppressors. In this context, it is important to consider the function of specific Rho GTPases and overlapping between members within the family (compensation), as well as the specificity of each individual protein in different cell types.

A plethora of in vitro studies demonstrates the indispensable function of Rho proteins for cytoskeleton dynamics within different cell types. However, only in recent decades we have got access to cell-specific genetically modified mouse models to decipher the complex regulation between members of the Rho family within diverse cell types in vivo. Translationally, alterations of expression and/or function of Rho GTPases have been associated with several pathological conditions, such as inflammation and cancer. Recent studies demonstrate that Rho GTPases play important roles for the maintenance of tissue homeostasis in the gut. We will summarize the current knowledge on Rho protein function within individual cell types in the intestinal mucosa in vivo, with special focus on intestinal epithelial cells and T cells, as two critical players for the maintenance of gut tissue homeostasis.

## 2. Rho GTPases

The actin cytoskeleton is a major cell structure, which is involved in multiple biological functions. The cytoskeleton is responsible for the maintenance of cell shape and cell mechanical resistance to deformation, while its association to the Extracellular Matrix (ECM) allows tissue architecture. Various proteins and factors regulate the dynamic structure of the actin cytoskeleton primarily. Belonging to the Ras family, Rho GTPases are known as important molecules involved in cytoskeletal reorganization [35], thereby coordinating diverse cellular functions, such as vesicular trafficking, cell cycle, cell polarity, and transcriptomic dynamics, also participating in key biological processes, such as cell morphology, proliferation, differentiation and migration. They all share a G domain, which enables GTPase and nucleotide exchange activity. Based on biological function and structural motifs, the Rho family can be divided into eight subfamilies: Cdc42, Rac, Rho, RhoBTB, RhoF, RhoH, RhoUV and Rnd [36] (Table 1). However, the most commonly studied ones are the Rho, Rac and Cdc42 subfamilies. Atypical Rho GTPases, such as Rnd, RhoH, Wrch-1, Chp or RhoBTB, are structurally different from the other Rho-family members. For instance, proteins belonging to the RhoBTB subfamily consist of a GTPase (guanosine triphosphatase) domain, a proline rich region, a tandem of two BTB (broad complex, tramtrack, and bric-a-brac) domains and a carboxyl terminal BACK (BTB and C-terminal Kelch). Rho GTPases are best known for their roles in the control of cytoskeletal events.

**Figure 1:**
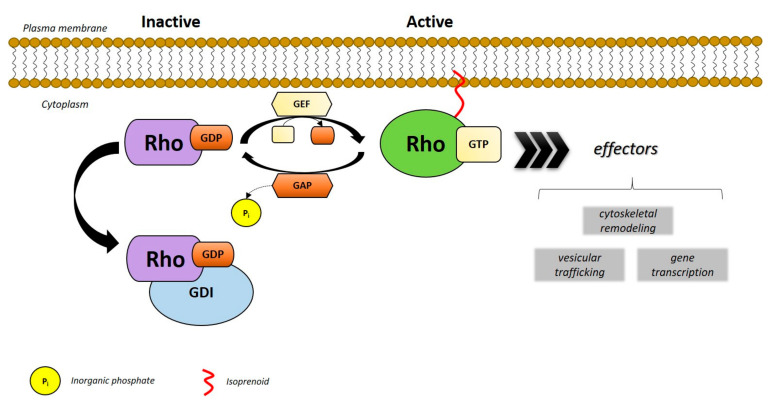
Activation of Rho proteins. Rho GTPases are known to act as “molecular switches” that cycle between an inactive (guanosine diphosphate-bound, GDP) and active (guanosine triphosphate-bound, GTP) forms, which are regulated by guanine nucleotide exchange factors (GEFs) and GTPase-activating proteins (GAPs). In their active form, Rho GTPases bind to effector molecules to generate a downstream response. Post-translational modifications, like the attachment of isoprenoid groups by geranylgeranyltransferases (GGTases), namely prenylation, allowed the proteins been targeted to the plasma membrane.

The activity of Rho GTPases havs been long considered as regulated by the switching between an active GTP- and an inactive GDP-bound form (Figure 1). This cycling is regulated by three types of regulatory proteins. Rho guanine nucleotide exchange factors (RhoGEFs) catalyze the exchange of GDP for GTP to activate Rho proteins [53]. Rho GTPase- activating proteins (RhoGAPs) increase the hydrolysis of intrinsic GTP, thereby inactivating Rho GTPases [54]. Rho guanine dissociation inhibitors (Rho GDIs) bind to Rho-GTPases and sequester them in the cytosol, thereby controlling their spatiotemporal activity [55]. However, considering atypical GTPases, 10 members of the family do not follow the classical GTPase cycle (50% of the family), and are generally not regulated by GTP-GDP cycling [56,57] and therefore do not require GEFs and GAPs [58]. Mechanisms aside from GTP-GDP cycling also influence Rho GTPase signaling. Ultimately post-translational modifications (PTMs) are responsible for the regulation and signaling of these GTPases [59].

Rho GTPases are regulated by a wide range of PTMs, which act together to achieve a proper function of these proteins in an appropriate spatiotemporal manner. Among them, protein prenylation has a vital role in determining the subcellular localization of Rho GTPases. C-terminal prenylation is the most frequent PTM of Rho GTPases, which includes the addition of a geranylgeranyl (20-carbon chain) or farnesyl (15-carbon chain) isoprenoid moiety to a Cys residue in the CAAX subunit of the protein. In general, prenylation is catalyzed by geranylgeranyltransferases (GGTase-1 and RabGGTase) and farnesyltransferase (FTase), respectively [59]. Based on its structure, most Rho GTPases are subjected to GGTaseI/GGPP-mediated prenylation, although FTase-mediated compensatory prenylation of Rho proteins has been described. Prenylated proteins can then be submitted to endonuclease (RCE1) [60] and carboxymethyltransferase (ICMT) [61] activity for post-prenylation processing. Along with prenylation, phosphorylation, sumoylation and ubiquitinylation are also crucial for the modulation of Rho GTPase activity [62,63].

Based on biological ubiquity, crosstalk between Rho proteins within the family is common. This is illustrated by the two most studied family members, namely RHOA and RAC1. The interconnection between these two proteins has been shown to coordinate a vast cluster of biological processes such as cell adhesion, membrane traffic, cell migration and division [64]. Although in some pathways both proteins are simultaneously activated, in many cases, either RHOA or RAC1 seems to be triggered, whereas the other is inhibited. For instance, during cell adhesion, suppression of RAC1 activity depends on the activation of the RHOA effector protein ROCK [65]. Therefore, a deep understanding of the interactions between different Rho proteins, and how individual Rho GTPases can affect each other’s expression and function, will be interesting to explore.

## 3. RHOA (Ras Homology Family Member A)

Active RHOA interacts with specific downstream effectors to trigger different cellular responses; therefore, RHOA controls actin–myosin contraction (ROCKI, ROCKII) [66], actin polymerization (ROCKI, ROCKII, DIA1, DIA2) [67] and actin remodeling (PRK2) [68]. Rho kinases (ROCKs) are the best known of Rho target proteins, which promote phosphorylation of the regulatory myosin light chain. Numerous publications describe a role of RHOA and ROCK-mediated phosphorylation of MLC2 in control of epithelial cytoskeleton by using in vitro approaches [69]. Besides the epithelium, RHOA plays a primary functional role in various innate immune cell types, namely macrophages, neutrophils, and dendritic cells (DCs); upon activation by pathogen-derived signals, these cells migrate towards the site of infection, where they help to internalize pathogens [70]. In the context of adaptive immune responses, RHOA function determines T-cell development in the thymus, as well as T cell migration [17].

### 3.1. RHOA Function and Intestinal Inflammation

Dysregulation of Rho protein function in the intestinal epithelium is associated with cytoskeletal dysfunctions [71]. In the GI tract, Segain et al. observed increased activation of RHOA in inflamed colonic mucosa from mice subjected to TNBS-induced colitis compared to control mice, which could also be confirmed in colonic biopsy specimens of CD versus control patients [72]. Nevertheless, the authors of this study did not analyze cell-specific modulation of RHOA activity within single cell types within the gut mucosa, since they assessed RHOA activation in whole tissue specimens. Recently, papers taking advantage of cell-specific genetic in vivo models contributed to set light in this context, validating the importance of RHOA protein in various cell types in the context of inflammation.

Various in vitro studies have demonstrated that Rho-mediated actin cytoskeletal modifications are critical for intestinal barrier function [73], since Rho and ROCK play a key role for the maintenance of TJs upon activation of various intracellular signaling pathways [74]. Both up and downregulation of Rho protein function can alter actomyosin contractility and leads to impaired barrier function [71,73]; thus, whether RHOA inhibition, activation or a combination of both would modify epithelial integrity and permeability still remains unclear [71]. Moreover, apoptotic as well as physiological epithelial cell extrusion requires Rho-mediated contraction of an intercellular actin/myosin process [75,76]. This clearly impacts on the maintenance of epithelial cell numbers, and therefore, tissue homeostasis.

Little is known about a potential modulation of RHOA expression within intestinal epithelium under inflammatory conditions in IBD, and available data are controversial. For instance, a combined in vivo/in vitro study suggested that ROCK inhibition reinforces barrier function via the upregulation of occludin in the epithelium, proposing ROCK as a potential target for patients with necrotizing enterocolitis, a neonatal form of IBD [77]. In contradiction, in our study we have shown that genetic deletion of *Rhoa* or *Pggt1b* (gene encoding for the prenylation-catalyzing enzyme geranylgeranyltransferase-I (GGTase-I) β-subunit) in murine IECs, impacted on cytoskeleton regulation and epithelial cell shedding, leading to severe epithelial permeability and gut inflammation. Interestingly, inflammation in IBD patients is correlated with cytosolic accumulation of RHOA within IECs, indicative of RHOA inhibition [78]. This discrepancy can be explained by the fact that ROCK is not only activated downstream of RHOA, but also under the control of other GTPases, such as RHOB or RHOC. This controversy is also supported in studies focusing on RHOA-related factors. It has been found that Rho-GDP dissociation inhibitor alpha was up-regulated in CD and UC patients [79]. Moreover, RhoGTPase-activating protein 17 (Arhgap17) participates in the maintenance of tight junctions and vesicle trafficking; mice carrying ARHGAP17-deficient IECs have shown an increased paracellular permeability and defective localization of the apical junction, although this is not causing spontaneous colitis, but increased the sensitivity towards DSS-induced colitis [80]. Taking together, RHOA can be considered as a crucial regulator for the maintenance of epithelial integrity and homeostasis in the gut. Nevertheless, it is still unclear whether RHOA inhibition or activation impairs epithelial leakage and permeability.

Numerous in vitro studies demonstrated that RHOA is important for T-cell activation and migration [81]. Additionally, thymus T-cell development is clearly affected by RHOA function. T-cell-specific *Rhoa* conditional knockout mice (using CD2-Cre or Lck-Cre deleters) proved that absence of RHOA leads to defective thymocyte β-selection, decreased thymocyte proliferation and survival [82]. Using similar models, Hasseldam and his co-workers have shown that the lack of RHOA in T-cells results in reduced numbers of mature T-cells in thymus and spleen but normal counts in peripheral blood, and are protected against experimental multiple sclerosis, hence RHOA is essential for the activation and migratory ability of T cells [83]. In our recent study, we confirmed alterations of total numbers of T cells in the periphery of T-cell specific *Rhoa* deficient mice, using in this case a CD4Cre eraser. Moreover, we have shown that *Rhoa*ΔCD4 (and *Pggt1b*ΔCD4) mice developed a spontaneous colitis due to increased expression of integrin α4β7 on T cells, which regulates their localization to the intestine [84]. Using a conditional knock-out of *Fam65b*, an atypical inhibitor of the small G protein RHOA, it has been revealed that T cell-specific deletion of *Fam65b* (CD4Cre) goes along with alterations of T cell migration in a RHOA-dependent mechanism [85]. Jointly, recent in vivo studies confirmed the physiological function of RHOA in T-cell development, migration and effector function.

### 3.2. RHOA Function and Cancer

Due to the critical functions of RHOA in cell proliferation, migration and apoptosis, it is expected that RHOA dysfunction is also associated with cancer. RHOA overexpression was observed frequently in many metastatic cancer cell types [86] and cancer tissues, and was first found to be upregulated in human colorectal tissue in 1999 [34]. Analyzing the gene profiles of 137 colorectal tumor samples in humans, it was indicated that *RHOA* is one of the most altered gene among *RAS* homologues, and RHOA expression shows a positive correlation with survival time [87]. Beside this, RHOA was found to be upregulated in chemoresistant-CRC, playing a role in the expression of cell membrane transporter and apoptosis in colon cancer, contributing to chemotherapeutic resistance in colorectal cancer patients [88].

The RHOA activator (Lysophosphatidic acid LPA) was found to induce colon cancer cell proliferation through β-catenin signaling, while protecting them from apoptosis through ERK, BAD and BCL2 [89]. Accordingly, reducing RHOA expression in colon cancer cells could considerably inhibit cell division and invasion, in a xenograft model [90]. The loss of epithelial polarity is associated with epithelial-mesenchymal transition, which is found in aggressive tumors. In vivo, RHOA deletion within IECs affects the expression of epiregulin (EREG) in the crypts, and thereby modulates the YAP-Hippo pathway, resulting in alterations of cell polarity and cytoskeletal organization [91]. IEC-specific RHOA KO mice showed remarkable loss of proliferative cells (ki67 and phospho-histone H3) throughout the whole intestine, together with increased arrested-mitotic and apoptotic cell number (cleaved caspase-3-positive) [91]. In conclusion, these studies suggested RHOA as a potential tumor-promoting factor in colon cancer, with a fundamental role in supporting tumor growth and invasiveness.

In agreement with the controversy about RHOA function within epithelial cells mentioned above, two recent studies suggest that RHOA function in CRC acts under a completely different mechanism as in other solid tumors, and inactivation of RHOA can also promote tumor progression. A study in 2014 showed that the activation of Wnt/β-catenin signaling through RHOA inactivation can accelerate tumor development [32]. In this study, RHOA inactivation in the intestinal epithelium of APCMin mice resulted in significantly more tumors and lower survival rate compared to control mice. Moreover, *Rhoa* was identified as one of the most differentially expressed genes in bad versus good prognosis CRC tumor samples; in this case, reduced *Rhoa* expression was correlated with shorter survival [87]. Hence, also in the context of cancer, it is not clear whether RHOA activation within IECs should be considered as a protective or harmful mechanism.

Genome-wide association studies (GWAS) have enabled the mapping of genetic alterations in CRC, at least in sporadic tumors [92]. Recent studies have shown that the overlapping between colitis-associated cancer (CAC) and sporadic CRC is only partial. Both entities carry similar somatic alterations and share common tumorigenesis pathways, but some discrete features exist between these two [93]. For instance, Wnt pathway mutations (including inactivation of *APC*, activation of *CTNNB1*) and *K-RAS* mutation, which are thought to be the most common alterations in sporadic CRC, appeared much less in IBD-CRC. Other mutated genes like *SOX9* and *EP300*, *NRG1*, *IL16* in contrast, appeared more frequently in IBD-CRC tumors. Interestingly, mutations in Rho- and Rac- pathway related genes (*DOCK2*, *DOCK3*, *PREX2*, *RADIL*) and other somatic mutations that activate/inactivate Rho GTPases were identified in 50% IBD-CRC, suggesting that non canonical WNT signaling can be a potential target in IBD [93]. This implied that RHOA can work differently in CAC, which opens the need of the study of RHOA specifically in CAC.

As mentioned above, RHOA was found to be a key element in efficient T-cell polarization and migration. In cancer, the Gly17Val *RHOA* mutation, which blocks the binding of GTP under guanine nucleotide exchange factor (GEF) stimulation, was found in all type of T-cell lymphoma, especially in angioimmunoblastic T-cell lymphoma (AITL) (53.3% or 68% [94,95]) and did not appear in any diffuse large B-cell lymphoma (DLBCL). Although Gly17Val *RHOA* mutation had no correlation with chemotherapy response or tumor development, it showed slightly different survival rate between non-mutant and mutant patient groups. Interestingly, *TET2* mutations, which is frequently found in hematologic malignancies, was also found in all cases of p.Gly17Val *RHOA* mutation [95], suggesting a strong correlation between *RHOA* and *TET2* dysfunctions. In an ulterior publication using Tg*RHOA*, it was revealed that CD4-mediated expression of *RHOA*G17V is sufficient to dysregulate T-cell development and confer autoimmunity, while the combination of *RHOA*G17V and *TET2* mutations lead to the development of lymphomas [96]. Despite the fact that RHOA may play certain role in T-cell biology in lymphomas, the involvement of RHOA in T-cell function in the context of CRC/CAC has not been explored to date to our knowledge.

### 3.3. Targeting RHOA in the GI Tract

Several recent publications support the potential of RHOA targeting in the context of intestinal inflammation. For instance, DSS-induced inflammation is partially rescued upon treatment with oxymatrine, which inhibits RHOA/ROCK leading to ameliorated epithelial barrier function and balanced cytokine secretion from Tregs/Th17 [97]. In humans, increased expression of leptin receptor OBR in the mucosa of UC patients is associated with RHOA activation, while deficiency of OBR in mice protects against TNBS colitis in mice [98]. Paradoxically, miR-31-3p protects against TNBS and DSS-induced colitis upon activation of RHOA [99].

Although controversial regarding promotion or blocking of the pathway, our current knowledge supports the exploitation of RHOA function in the context of CRC. Thus, RHOA regulating proteins have been suggested as biomarkers in CRC metastasis, such as ARHGGAP5 [100] and ARHGAP10 [101]. In humans, activation of RHOA in different cell types promotes CRC progression; this is the case of upregulated expression of GPR4 in the TME [102], or GEF-H1 in total tumor tissue [103]. On the other hand, CXCR4 promote cancer progression in APCMin mice by causing cytoskeleton alterations and recruitment of immune cells, in a RHOA dependent mechanism [104]. In line with a role for RHOA inactivation in CRC, a recent publication demonstrates that upregulation of IRX5 in CRC goes along with inflammation (CXCL1, CXCL8 expression) and inactivation of RHOA/ROCK1/LIMK1, which correlates with poor prognosis [105].

## 4. RAC1 (Ras-Related C3 Botulinum Toxin Substrate 1)

RAC1 is one of the best described members among Rho GTPases; many *RAC1* mutations were introduced into in vitro and in vivo models in order to characterize this protein. In general, Rac proteins play important roles in apoptosis in intestinal epithelial cells, barrier function via cytoskeleton regulation, ROS production and leucocyte trafficking. RAC1 controls actin polymerization and lamellipodia formation, as well as actin turnover [106]. RAC1 takes part in many cellular pathways such as PAKs, NFkB, MAPKs, Wnt/β-catenin, STAT3-, and its downstream products regulate various cell activities like cytoskeletal management, cell–cell contacts, cell polarity, cell migration, transcription, proliferation, etc. However, the effect of RAC1 is often debatable, promoting some pathological but also protective mechanisms, indicative of an overlap between physiological and pathological functions.

### 4.1. RAC1 and Intestinal Inflammation

The alteration of RAC1 activity has been shown to be associated with immunodeficiency. RAC1 is a key protein in many inflammation-related processes including apoptosis, intestinal barrier function by cytoskeletal regulation and reactive oxygen species (ROS) generation. Its role is important in a wide variety of cell types, including intestinal epithelial and immune cells. Therefore, RAC1 keeps critical roles in the maintenance of epithelial homeostasis under both physiological and pathological conditions, but also is a potential therapeutic target against immune diseases. In the context of the GI tract and gut-related pathologies, the crucial role of RAC1 is clearly evidenced by the use of azathioprine in the current treatment of IBD. Azathioprine-mediated immunosuppressive effect is based on the RAC1 inactivation on T cells, leading to the induction of apoptosis [107]. In the context of intestinal inflammation, upregulation of RAC1 has been found in whole colon tissues upon inflammation, both in mouse DSS colitis and whole colonic tissue from UC patients [108]. *RAC1*, but also *CDC42*, are suggested as genes which are differentially expressed in adult and pediatric UC [109]. Some studies suggested that RAC2 is also associated with human IBD; nevertheless, its role in disease pathogenesis is still unclear. *RAC2*^−/−^ mice suffer from a severe disease when submitted to a C.rodentium-induced infectious colitis model, which suggest that impaired RAC2 function is important in regulation of epithelial function and could potentially be involved in human IBD [110].

Several in vitro and in vivo studies revealed that genetic deletion of RAC1 within intestinal epithelium are associated with defects on epithelial cell proliferation, migration and/or differentiation, with important implications for epithelial homeostasis [111]. Myant et al. demonstrated that RAC1 is sufficient to drive stem cell proliferation and tissue regeneration in response to damage [112], while Stappenbeck et al. showed that *RAC1* mutations originates defects in epithelial cell differentiation in vivo [113]. A recent study from Sumigray et al. described the role of *RAC1* for compartmentalization of crypts and villi in the small intestine of neonatal mice. Lack of RAC1 within IECs (*Rac1*Villin-CreERT2 mice) impair cell shape alterations at the interface between crypt and villus, and the abrogation of hinges, affecting tissue architecture [114]. This publication confirmed the relevant role of RAC1 in vivo for epithelial cell morphology and maintenance of tissue architecture. However, the fact that they use neonatal mice implies that there might be some differential features, in terms of epithelial maturation and colonization with the microbiota. It would be important to compare these observations to the effect of *Rac*1 deletion in the adult intestinal epithelium.

Rac proteins are likely to play an important role in T-cell biology. In the GI tract, genetic alterations on genes encoding for Rac proteins leading to increased Rac activity in peripheral blood cells have been associated to IBD [115,116]. Azathioprine is one of the oldest immunosuppressive drugs used for the treatment of various autoimmune and chronic inflammatory diseases such as rheumatoid arthritis or IBD [117]. It has been demonstrated that azathioprine and its metabolite (5-MP) induce apoptosis in T cells from CD patients by modulating RAC1 activation, upon CD28 co-stimulation [107]. Azathioprine response is shown in *RAC1* WT IBD patients, versus patients carrying *RAC1*1 mutations [118], and patients with active disease which respond to azathioprine therapy show decreased RAC1-GTP and RAC1 expression [119], suggesting that RAC1 could be used as biomarker of azathioprine response. Another member of the Rac family is RAC2, which is specifically expressed in hematopoietic and endothelial cells, and is responsible for cell integrin and immune-receptor signaling [120,121]. Many reports also indicated that knockdown of *Rac2* in mice had significant effects on immune cells, for example B cell development and signaling [122], T cell differentiation [123] and activation [124], T cell distribution and chemotaxis [125]. Mice deficient in both GTPases *Rac1*flox/floxCD2-Cre x *Rac2*(^−/−^) show discernible effect on the number of peripheral T cells, while either *Rac1* or *Rac2* alone had no effect on thymic T-cell development [126]. This is in agreement with our own observations, since total numbers of T cells in the thymus and the periphery were not altered upon *Rac1* deletion within T cells [84].

### 4.2. RAC1 and Cancer

Considering its function in various cellular processes, RAC1 appears as a potential oncogenic target. In different organs, RAC1 is required for full oncogenic transformation of RAS in vitro [127] and in vivo [128,129]; it promotes neovascularization and maintains cadherin-mediated cell–cell contacts. In human CRC, although the *RAC1* mutation rate was relatively low compared to other cancer types like skin cancer, the overexpression of RAC1 protein was noticed and it tightly linked to tumor stage and metastasis [130]. Metastasis represents the main cause of colorectal cancer-related death, and according to 2018 cancer statistics, metastasis occurred in 50% of colorectal cancer patients in America even after surgery [131], while liver metastasis accounted for 10% to 25% among that [131,132]. RAC1 protein expression in liver metastatic tissue was higher than in colon tumor itself, and the higher the expression of RAC1, the shorter the survival time [133]. In 2018, a meta-analysis study combining results from 1793 patients indicated that positive RAC1 expression does not relate to histological differentiation but associates with tumor stage, vessel invasion and lymph metastasis [134].

Epithelial-mesenchymal transition (EMT) is an early step of cancer cells in which the cell transforms to acquire an invasive phenotype. Overexpression of *RAC1* in colon cancer cells induced an aberrant expression of EMT markers (vimentin, N-cadherin, E-cadherin) and promoted invasion, migration and metastasis in vitro and in vivo [135]. In addition, RAC1 was found to be upregulated in the intestine of APCMin mice, while *Rac1* deficiency could impair progenitor cell over-proliferation and diminished the transformation of Lgr5^+^ stem cells in APC-mutated crypts [136]. Moreover, the lack of *Rac1* impeded tumor formation as well as prohibit *Kras*-mutant tumor development, suggesting that RAC1 could be a potential curative target for initial colon cancer stage. Similarly, RAC1b—a constitutively activated isoform of RAC1—was found to be upregulated in colon cancer tissues, and emerged as a potential target to overcome chemotherapy resistance in colon cancer. RAC1b overexpression caused cancer cell hyperproliferation via NF-κB pathway, while blocking RAC1b could reduce cell division in vitro and in vivo [137]. The role of RAC1b for wound-healing after resolution of inflammation and, in turn, for tumor promotion was confirmed in another study. Ectopic expression of RAC1b within IECs enhanced APC-dependent intestinal tumorigenesis, but alleviated inflammation-dependent tumorigenesis in the AOM/DSS model [138]. These observations are interestingly in agreement with the previously mentioned differential signatures between CAC and CRC, and support the hypothesis that RAC1/RHOA pathway might be subjected to a different regulation in colorectal cancer in IBD patients [93].

The integrity of RAC2 is indispensable for hematopoietic malignancies. Despite the fact that knocking down either *RAC1* or *RAC2* is adequate to reduce the transformed MLL-AF9 leukemia development; RAC2 but not RAC1, plays a dominant role in the initiation of acute myeloid leukemia in vivo [139]. RAC2 was found to positively regulate tumor progression through controlling macrophage M1 to M2 differentiation and metastasis in the mice model [140]. Similar to RHOA, to our knowledge the role of Rac proteins within T cells in the context to CRC/CAC has not been explored so far.

### 4.3. Targeting RAC1 in the GI Tract

Recent reviews collected strategies meant to target RAC1 function in different cell subtypes in the context of inflammation, and IBD [117]. Highly relevant, RAC1 inhibition as being suggested as biomarker of thiopurine therapy [119]. In this context, it is important to consider multicellularity in the intestinal mucosa, and the potential effects of thiopurines on other cell types, beyond T cells. For instance, 6-MP targeting of NK cells, has been previously neglected [141], and might be exploited in IBD. Important as well is the RAC1-mediated cytoskeleton regulation within IECs. Modulation of Rac signaling may provide interesting pharmacological opportunities to design specific drugs for epithelial restoration. In this context however, recent studies suggest that activation rather than blocking of RAC1 might be exploited in epithelial restoration. Interestingly, ELMO1 protects against DSS-induced colonic injury in mice through its effects on epithelial migration via RAC1 activation [142]; PAF increases healing of mucosal wounds via RAC1 pathway [143]. An important step towards targeting RAC1 for therapy is the specific targeting, interfering uniquely with RAC1 signaling.

RAC1 appears as an attractive candidate in the context of malignancies. Taking advantage of in vivo experiments, it was shown that thiopurines treatments (TG) inhibited CAC in the AOM/DSS mouse model, via decreased β-catenin in a RAC1-dependent mechanism [144]. In the context of epithelial cell targeting, EMT and metastatic potential might be inhibited upon activation of RAC1; for instance, upon CSRP2 treatment (Hippo-ERK-PAK/LIMK/cortactin) [145], miR-142-3p transfection [146], upregulation of SSH3 (LIMK) [147] or PLS1 (ERK1/2) [148], downregulation of DMTN [149], or IRF1 suppression [150]. Recent work from An et al. revealed the different behavior of RAC1 inhibition in combination with irradiation on cell cycle. Radiation treatment together with RAC1 targeting (NSC27366) protected normal intestinal cells, but not tumor cells, by supporting cell cycle and reducing ROS production remarkably. In contrast, similar treatment caused significant enhanced tumor cell apoptosis [151]. These observations underscore the relevance of RAC1 inhibition in the context of intestinal radiotherapy.

## 5. CDC42 (Cell Division Control Protein 42)

CDC42 is another player within RhoGTPases, has a significant role in the regulation of actin dynamics, polarity and paracellular permeability [152]. Similar to other Rho family members, under the regulation of interacting factors (GEFs, GAPs, GDIs), CDC42 switches between inactivate and activate cycle to stimulate various cellular processes, with the most and initial known function is to regulate cell migration and polarity via actin remodelling [153]. CDC42 is involved in sensing the direction of cells by regulating the filopodia formation [154] through N-SWASP, in fibroblasts. Moreover, it is an essential factor for the regulation of epithelial cell polarity, distinguishing apical membrane (facing the lumen) and basal membrane (connecting to cellular matrix) [155], but also stem cell proliferation.

### 5.1. CDC42 and Intestinal Inflammation

In the context of human intestinal inflammation, the expression of CDC42 is decreased in active IBD, and *CDC42* has been suggested as a target of MiR-15a for the regulation of epithelial junctions (ZO1 and E-cadherin) in pediatric patients [156]. In contrast, another study describe the upregulation of ACK1 (Activated CDC42 kinase1) in colitis as well as colorectal dysplasia [157].

Various in vitro studies have demonstrated that CDC42 controls various cellular functions within epithelial cells, such as adhesion, and migration [155]. Furthermore, CDC42 is critical for intestinal stem cell division, survival and differentiation, as well as the formation of a functional intestinal barrier. Two recent publications suggested that the lack of CDC42 within IECs results in alterations of epithelial architecture (microvillus inclusion) and impaired cell differentiation. In the first report, CDC42-deficient mice (*Cdc42*loxP/loxP; VillinCre) elicit increased numbers of mucin and chromogranin A^+^ cells while remarkably decreased numbers of stem cell and Paneth cells. Additionally, *Cdc42* deletion at stem cells showed abnormal cell proliferation and apoptosis, defected clonal expansion capacity and Paneth cell differentiation [158]. On the other hand, another group could demonstrate that the same animal model shows epithelial cell polarity defects, and suffer from a pathology similar to microvillus inclusion, with microvesicle accumulation in the intestinal epithelium. Likewise, CDC42 deficiency affects Paneth cell differentiation and localization, causing epithelial architecture alterations and disrupted intestinal permeability [159]. In the context of mouse experimental colitis, overexpression of CDC42 through injection of corresponding adenovirus vector, resulted in reduced levels of the cytokines IL-10, IFN-γ, IL-4, and TNF in TNBS-treated mice [160].

CDC42 also plays an essential role in human T-cell development and effector function [161]. Guon et al. took advantage of conditional knockout mice of *Cdc42* utilizing Mx1-Cre mice to demonstrate that CDC42 is required for thymopoiesis and effector/memory T cell differentiation. They observed that deficiency of CDC42 blocks thymopoiesis and induces enhanced naive T cell differentiation to effector and memory cells, which plays a restrictive role in autoimmunity [162]. Interestingly, in our study we have shown that *Cdc4*2ΔCD4 mice exhibited decreased numbers of CD4^+^ and CD8^+^ T cells in blood, spleen, and mesenteric lymphoid nodes, supporting the essential role on T cell homeostasis. However, we could not mimic the intestinal phenotype observed in mice carrying RHOA-deficient T cells, at least under basal conditions [84]. Using a different Cre-deleter, it was shown that mice lacking CDC42 in T cells (*Cdc42*-LCKCre) have enhanced Th17 differentiation and suffer from a wasting disease in mouse models of colitis, causing a fatal lymphoproliferative disease [163]. Thus, CDC42 impairs Th17 differentiation, and maintains the balance between Th17 and Tregs. We assume that the use of different deleters and the model affecting thymus T cell development might explain these controversial data.

### 5.2. CDC42 and Cancer

Many studies have indicated that CDC42 activation is associated with oncogenesis [164]. In general, the movement of intestinal epithelial cell depends on direction, lamellipodial protrusion, adhesion formation and cell polarization; thus, it was also proven that CDC42 modulates directly colorectal cancer cell invasion in vitro [165]. For example, a *CDC42* cycling mutation (*Cdc42*Hs(F28L)) in fibroblasts could activate the c-Jun kinase (JNK1) and stimulate filopodia formation, cause several changes in cell behaviour, which likely contribute to promote cellular transformation [164]. Accordingly, the overexpression of CDC42 was observed in many cancer tissues like thyroid, lung, head and neck, stomach, pancreatic, breast, colon, etc. CDC42 was highly expressed in 60% of human colorectal cancer even if no mutation has been detected [166]. PAK5, a CDC42/RAC1-dependent activated kinase is upregulated in CRC versus adjacent tissue, and this is correlated with cancer progression [167].

Adenomatous polyposis coli (*APC*) mutation is referred as an initiator of colorectal cancer [92], it frequently occurred (81%) in the non-hypermutated colon and rectum cancer. APC-stimulated exchanging factor (ASEF1 and ASEF2) activities were found to be CDC42 specific, and not RAC1-dependent [168]. ASEF suppressed cell malignant transformation by impairing APC-dependent CDC42 activation [169]. In agreement with the role of CDC42 for intestinal stem cell homeostasis, less than 1% of *Cdc42*-KO crypts survived up to 72 h in culture in intestinal organoid cultures. Indeed, *Cdc42* ablation in tumor cells suppresses progression of tumors [170]. In agreement with the role of CDC42 in cell migration and movement, overexpression of *Cdc42* induced APC^Min/+^ intestinal tumor progression in mice; consequently, inhibiting CDC42 activity in β-catenin–mutant mice could lead to intestinal tumorigenesis suppression [170]. Together, CDC42 is proved to be involved in tumor promotion.

Despite the role of CDC42 within tumor cells in general and IECs in the context of CRC, little is known about immune cells and other cells within the tumor microenvironment and CDC42-mediated regulation of their functions in cancer. In fibroblasts, CDC42 activation contributes to tumor promoting activity of cancer-associated fibroblasts (CAFs), and impacts on matrix remodelling or angiogenesis [171]. In the context of chronic lymphocytic leukemia, CDC42 upregulation, together with downregulation of RAC1 and RHOA, upon direct contact with tumor cells, impairs migration of T cells and therefore immunosurveillance [172]. As in the case of Rho GTPases, the lack of studies analyzing the role of CDC42 within T cells in the context of immunosurveillance in CRC impairs drawing any conclusion in this perspective.

### 5.3. Targeting CDC42 in the GI Tract

Not much is known about targeting CDC42 in the context of intestinal inflammation. A recent publication describes the mechanism by which HuR (RNA-binding protein HuR) promotes protein expression of CDC42 within IECs, contributing to epithelial restitution (healing) in ischemia as well as colitis models (DSS), in a mechanism that is dependent on the actin cytoskeleton [173]. As mentioned above, MiR-15a regulates epithelial junctions via CDC42-dependent mechanisms in pediatric IBD patients [156].

In contrast to the role of CDC42 in inflammation, the well-established effect of CDC42 in tumor promotion paved the way for the description of different targeting strategies. Suggested as a cancer biomarker, a recent publication indicated that the concomitant expression of CDC42 and CACNA2D2 shows improved power as CDC42 alone, in the context of CRC diagnosis [174]. Different factors, which have been described to be upregulated in CRC, are associated with activation of CDC42, as a mechanism promoting tumor progression. For instance, POTEE is upregulated in CRC, and it promotes cancer cell proliferation and tumor growth and metastasis in vivo by activating RAC1 and CDC42 [175]. MiR-20a/miR-106a promotes the loss of WTX, which impairs the interaction between CDC42 and Rho-GDIα, and therefore promotes CDC42 activation, which contributes to cancer progression [176]. In agreement, targeting of molecules causing CDC42 repression successfully blocks tumor progression and/or metastasis. Targeting PAK5 impairs tumor cell migration and proliferation [167]. Overexpression of MiR-384 repressed the expression of K-Ras and CDC42, and impair invasiveness in vitro, and metastatic potential in vivo [177]. Another example is represented by VEGFR blocking, which goes along with changes in the subcellular localization of CDC42 which contribute to the control of the cancer [178].

## 6. Other Rho GTPases

As mentioned above, RHOA, RAC1 and CDC42 are the most studied members among Rho GTPases. However, recent studies show that other members of the family might also play a role in health and disease in general, and most specifically in the gastrointestinal tract. In this context, it should also be taken into account the interaction of classical Rho GTPases (RHOA, RAC/ CDC42) and these new members, as well as the potential functional compensation between them. Intimately connected to RHOA, RHOB and RHOC arose the attention of the scientific community in the last years. Furthermore, the atypical RHOU/WRCH1 related to CDC42/RAC1 appeared as a key player in cytoskeleton-mediated control of gut epithelial morphogenesis [179].

### 6.1. Inflammation

In the context of epithelial integrity, RHOB has recently emerged as an attractive protein. miR-21 KO mice are protected against DSS-induced colitis by simultaneously promoting RHOB and decreasing CDC42 expression, therefore improving epithelial integrity. This argues for a protective role of RHOB within IECs [180]. Interestingly [180], an independent study demonstrates that this mechanism can also explain the TJ impairment in UC patients due to downregulated expression of RHOB [181]. These studies suggest that RHOB plays a protective role in the context of epithelial integrity.

### 6.2. Cancer

Both RHOB and RHOC have been intensively studied in the context of malignancies and epithelial cells. Epithelial RHOB activation in response to several stress stimuli (DNA damage, hypoxia) counteracts tumor growth and cell migration/invasion, while promoting apoptosis [182], even in colon cancer cells [183]. Thereby, RHOB is suggested as a tumor suppressor. Accordingly, downregulated expression of RHOB has been observed in tumors [184]. On the other hand, the role of RHOC for promoting EMT, invasiveness [185] and vascularization [186] of tumors suggested this protein as target candidate in cancer and metastasis [187,188]. A correlation between RHOA and RHOC has been reported in the context of CRC [189], and can be targeted in vivo [90]. Likewise, lupeol induced RHOA and RHOC downregulation, impairing colorectal cancer cell invasion and migration [190], while targeting of Formin-like3 promotes CRC invasion in vitro [191]. The fact that some processes like EMT are reciprocally regulated by RHOA/RHOC [192] might explain the controversial data about RHOA blockade upon ROCK inhibition and selective targeting of RHOA. Beyond RHOB and RHOC, RNA editing of RHOQ [193] and hypermethylation of RAC3 are associated with CRC [194] and invasiveness in colorectal cancer cells.

Related to CDC42 and its role within the Wnt pathway, RHOU is normally expressed in the differentiated epithelium. Interestingly, a recent publication showed downregulated RHOU expression in human CRC. Mechanistically, the authors could demonstrate that abolition of RHOU expression within the intestinal epithelium caused a hyperplastic phenotype affecting all IEC subtypes associated with reduced apoptosis and increased proliferation, which also occur in *RHOU*-deficient tumor cell lines. Strikingly, inhibition of RHOU went along with increased RHOA activity [195].

## 7. Conclusive Remarks

Small GTPases belonging to the Rho family represent important mediators in the context of immunomodulation, mostly due to their key role in cytoskeleton rearrangement. Based on ubiquitous expression, Rho proteins are important in various cell types, which has been extensively demonstrated via in vitro studies. However, these studies could also show that the outcome of activation of single Rho proteins can be very different between discrete cell types, so it is important to define the contribution of each protein for every cell type within a determined tissue/system. This is also relevant in the context of functional compensation between Rho proteins. It is therefore important to consider the overlapping or antagonistic functions between different small GPTases, which contributes to the complexity of small GTPase function regulation. This is nicely exemplified by antagonistic functions between RAC1 and RHOA [115], but should not be limited to the most studied members of the family, and be expanded to the newly identified proteins. Another level of complexity is shown by the participation of several GAP/GEF in the regulation of Rho proteins, which sometimes are shared by several Rho proteins. Moreover, Rho GTPases are capable of interacting with different effector proteins, which would, at the end determine the cellular/molecular outcome of the downstream pathway activation, as well as the deregulation of small GTPase functions. All these levels of complexity in terms of its regulation should be taken into account in order to achieve working therapy strategies based on the modulation of Rho GPTase function.

**Figure 2:**
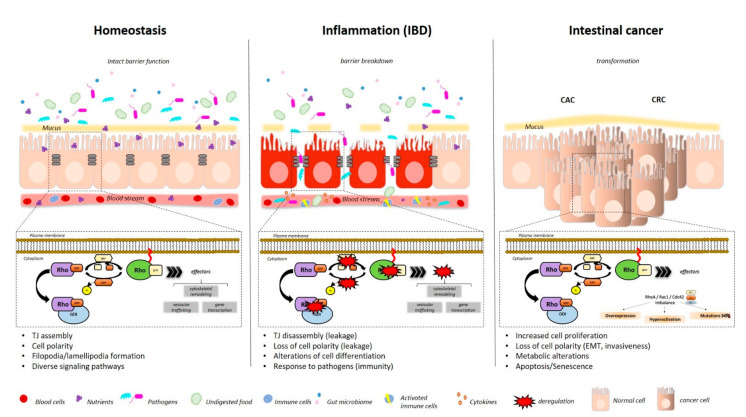
Alterations of small GTPases function within Intestinal epithelium in the context of Intestinal Bowel Disease (IBD) and Colorectal Cancer (CRC).

In vitro studies should set the basis for specific roles within single cell subtypes, but must be ultimately confirmed in in vivo studies, where the physiological situation in a multicellular system can be clearly tested. So far, the lack of cell-specific studies limits our knowledge about the effect on specific cell types in vivo, but this has significantly developed in the last decades. In this review, we have tried to summarize the current knowledge coming from in vivo studies describing the role of RHOA, CDC42 and RAC1 in the context of intestinal homeostasis and disease, focusing on cell-specific effects on IECs and T cells. As a summary, Rho GTPases are important for TJ assembly and cell polarity, impacting on epithelial architecture, integrity and differentiation in IBD, but also for cell transformation/EMT and invasiveness, as well as stem cell division, in the context of CRC (Figure 2).

In the case of adaptive immunity, Rho proteins regulate T cell development in the thymus (especially RHOA and CDC42), as well as T cell activation, differentiation and cytokine secretion. All this might be relevant in the context of intestinal inflammation. However, little is known about the role of Rho GTPases within T cells in the context of immunosurveillance in CRC. Beyond RHOA, CDC42 and RAC1, RHOB and RHOC emerge as promising candidates in IBD and CRC, based on in vitro data; this supports further research to confirm their role in physiological/pathological conditions in vivo. On the other hand, the role of RHOU for morphogenesis and homeostasis of the intestinal epithelium underscores the relevance of this atypical Rho GTPase. Moreover, we have tried to show exemplary preclinical data showing the potential of Rho GTPases as targets in IBD and CRC pharmacological management. Together, this text shows the potential, but also the complexity of Rho GTPases in the context of intestinal homeostasis and disease, and support basic and translational research in this context.

## Figures and Tables

**Table 1:** Rho GTPase (Guanosine triphosphatase) family of proteins. Rho GTPases are members of the Ras superfamily of small GTPases. They are small molecular weight proteins that play critical roles in many cellular processes. The Rho family consists of 20 members, categorized in 4 clusters and 8 subfamilies. Previous common names and symbols are based on the HUGO gene nomenclature committee.

	Cluster	Subfamily Name	Name	Approved Name	Alternative Name(s)/Symbol(s)	Original Reference
**Rho Family**	I	Rnd	RND1	Rho family GTPase 1	Rho6, ARHS, RHOS	Nobes, C.D. et al., 1998 [37]
			RND2	Rho family GTPase 2	ARHN, Rho7, RhoN	
			RND3	Rho family GTPase 3	ARHE, RhoE, Rho8	Foster, R.K. et al., 1996 [38]
		Rho	RHOA	ras homolog family member A	ARHA, ARH12, Rho12, RhoH12	Madaule, P. et al., 1985 [39]
			RHOB	ras homolog family member B	ARH6, ARHB, RhoH6, MST081	Cannizzaro, L.A. et al., 1990 [40]
			RHOC	ras homolog family member C	ARH9, ARHC, Rho9	
		RhoD/RhoF	RHOD	ras homolog family member D	RhoHP1,Rho, ARHD	Mrphy, C. et al., 1996 [41]
			RHOF	ras homolog family member F, filopodia associated	ARHF, Rif, FLJ20247	Ellis, S. et al., 2000 [42]
	II	Rac1/RhoG	RAC1	Rac family small GTPase 1	TC-25, p21-Rac1, Rac-1	Didsbury, J. et al., 1989 [43]
			RAC2	Rac family small GTPase 2	EN-7	
			RAC3	Rac family small GTPase 3	-	Haataja, L. et al., 1997 [44]
			RHOG	ras homolog family member G	ARHG, MGC125835, MGC125836	Vincent, S. et al., 1992 [45]
		Cdc42/RhoJ/ RhoQ	CDC42	cell division cycle 42	G25K, CDC42Hs	Polakis, P.G. et al., 1989 [46]
			RHOJ	ras homolog family member J	RASL7B, ARHJ, FLJ14445, TCL	Vignal, E. et al., 2000 [47]
			RHOQ	ras homolog family member Q	RASL7A, ARHQ, TC10	Neudauer, C.L. et al., 1998 [48]
		RhoU/ RhoV	RHOU	ras homolog family member U	ARHU, WRCH-1, DJ646B12.2, FLJ10616, WRCH1, CDC42L1, hG28K, fJ646B12.2	Tao, W. et al., 2001 [49]
			RHOV	ras homolog family member V	ARHV, Chp, WRCH2	Aronheim, A. et al., 1998 [50]
	III	RhoH	RHOH	ras homolog family member H	ARHH, TTF	Dallery E. et al., 1995 [51]
	IV	RhoBTB	RHOBTB1	Rho related BTB domain containing 1	KIAA0740	Rivero F. et al., 2001 [52]
			RHOBTB2	Rho related BTB domain containing 2	KIAA0717, DBC-2	
